# 

**Published:** 1903-12-12

**Authors:** 


### CONTENTS.

**The Arsenical Commission** - - - - - - 181
**The Basis of Professional Secrecy** - - - - 182
**Annotations** - - - - - - - - - 183
**Notes and News** - - - - - - - - 184
**Hospital Clinics and Medical Progress—**
What We Owe to Experiments on Animals - - - - - 185
The Administration of Chloroform - - - - - - 185
Traumatic Separation of the Lower Epiphysis of the Humerus - 186
The Operative Treatment of Retroversion and Retroflexion of the Uterus - - - - - - - - - - - 187
The Treatment of Appendicitis - - - - - - - 187
Progress in Disease of Digestive Organs - - - - 188
Progress in Bacteriology - - - - - - - - 189
Progress in Neurology - - - - - - - - 190
**The London School of Tropical Medicine** - - 192
**Hospital Administration—**
London Hospitals with Medical Schools and their Sites - - 193
Visits to Private Asylums - - - - - - - - 196
The Surgical Aid Society - - - - - - - - 197
"The Hospital" Library and Charities Bureau - - - - 198
**The Student of Medicine** - - - - - - - 198

### *NURSING SECTION.*

**Notes on News from the Nursing World—**

Christmas in Foreign Hospitals—Our Christmas Distribution—The Nurses' Coronation Fund—A Compliment to Irish Roman Catholic Nurses—The Acland Home and District Nursing—The Lady Superintendent of Prince Alfred Hospital, Sydney—The Suspension of a Night Nurse at Trim—The Refusal of a Correct Certificate—The Result of a Rash Experiment—District Nursing in Cheltenham—Male Nurses and the Royal Army Medical Corps—Action for Libel by a Handsworth Nurse—The Better Way - 137-139

**The Nursing Outlook** - - - - - - - 140

**Christmas in Foreign Hospitals—**

The Eppendorf Hospital, Hamburg - - - - - - 141

The Rigshospital, Christiania - - - - - - - 143

Berlin Municipal Hospital - - - - - - - - 145

Christmas and the Roman Hospitals - - - - - - 147

Christmas at a Government Hospital in Cyprus - - - 149

**"The Hospital" Convalescent Fund** - - - - 149

**The Evolution of the Modern Nurse** - - - - 150

**Association for Promoting the Training and Supply of Midwives** - - - - - - - 152

**Nurses' Missionary Union** - - - - - - 153

**Presentations** - - - - - - - - - 153

**To Help the Nurses to Help Themselves** - - 153

**Christmas Books** - - - - - - - - - 154

**Novelties for Nurses** - - - - - - - 155

**Appointments** - - - - - - - - - 156

**Everybody's Opinion** - - - - - - - - 157

**Notes and Queries** - - - - - - - - 158

